# \Interest of Hypnosis in Reducing Pain and Anxiety among patients scheduled for parietal urological surgery

**DOI:** 10.1192/j.eurpsy.2026.10790

**Published:** 2026-07-31

**Authors:** I. Kacem, A. Ghenim, M. Ajmi, C. Sridi, A. Gaddour, F. Chelly, I. Fki, M. Maoua, M. Kahloul, W. Naija

**Affiliations:** 1 Occupational Medicine and Professional Pathologies Department, Farhat Hached Teaching Hospital; 2 Research Laboratory LR19SP03, University of Sousse, Faculty of Medicine of Sousse,; 3 Anesthesia and intensive care department; 4 Occupational Medicine and Professional Pathologies Department, Sahloul Teaching Hospital, Sousse; 5 Occupational Medicine and Professional Pathologies Department, Ibn El Jazzar Teaching Hospital, Kairouan, Tunisia

## Abstract

**Introduction:**

Pain management remains a major challenge in perioperative medicine. Hypnosis has emerged as a promising alternative, capable of modulating pain perception, reducing anxiety, and improving the overall perioperative experience for patients.

**Objectives:**

To evaluate the perioperative benefits of hypnosis in the context of parietal urological surgery.

**Methods:**

This was a randomized, controlled, single-blind clinical trial conducted among patients scheduled for parietal urological surgery under general anesthesia in the urology operating room at Sahloul University Hospital of Sousse, over a four-month period (February to May 2025). The patients included in the study were randomly assigned to two groups of 15 patients each: Intervention group (Group I): patients who received a session of self-hypnosis immediately before anesthetic induction. Control group (Group C): patients who underwent general anesthesia without hypnosis. Postoperative pain and anxiety were assessed using the Visual Analog Scale (VAS) every 4 hours during the first 48 hours.

**Results:**

Preoperative anxiety VAS was 7.74 ± 1.19 in group I and 7.33 in group C (p = 0.423). Postoperatively, pain intensity decreased progressively in both groups. Mean VAS scores were comparable between groups I and C at all time points, except at H24, where the mean VAS was 0.06 ± 0.25 in group I and 0.73 ± 0.79 in group C (p = 0.007), showing a significant reduction in pain in the hypnosis group. Postoperative anxiety was overall lower in the hypnosis group, with significant differences observed at H4 (p = 0.048), H8 (p = 0.006), and H24 (p = 0.005).

**Conclusions:**

These findings suggest a beneficial effect of hypnosis, as it significantly reduces both postoperative pain and anxiety, thereby improving the overall recovery experience after surgery.

**Disclosure of Interest:**

None Declared

